# Assessing utility and completeness of information transmission during emergency department transfers

**DOI:** 10.1186/s12245-018-0203-x

**Published:** 2018-10-29

**Authors:** Jason J. Lewis, David W. Schoenfeld, Alden Landry

**Affiliations:** Department of Emergency Medicine, Beth Israel Deaconess Medical Center, One Deaconess Road, Rosenberg Building 2, Boston, MA 02215 USA

**Keywords:** Transfer documentation, Emergency department transfer, Medical data transfer

## Abstract

**Background:**

The transfer of patients from community emergency departments to tertiary care centers is a daily occurrence in the practice of emergency medicine, but the completeness of medical data in the transfer documentation is a relatively unstudied area. The goal of this study was to evaluate the completeness of information transmitted in the transfer documentation between transferring and accepting institutions and its perceived value at the receiving tertiary center on medical management.

**Methods:**

Prospective, observational, and convenience sample survey study at a tertiary referral center in Boston, MA.

**Results:**

A total of 100 surveys were completed during the 2-month study period. The presence of the radiology report and the provider note was most important in physician assessment of utility of the transfer packet for subsequent care of patients, yet these were the most commonly missing items (31.1% and 21% respectively). Other common missing data were medication administration records, nursing notes, and laboratory results.

**Conclusions:**

Medical data is absent in 15–31% of patients transferred from a community ED to a tertiary center. Provider notes and radiology reports were assessed as having the most utility to the receiving physicians.

## Background

The transfer of patients from one emergency department (ED) to another is a common occurrence. Typically, these occur from community hospitals to larger medical centers for specialized care. Federal laws, including the Emergency Medical Treatment and Labor Act (EMTALA), govern the logistics of transfers requiring direct communication between the sending and receiving hospitals, as well as sending relevant records and imaging. However, there is no standard for the transfer of patients’ clinical information between EDs.

While there have been a number of studies investigating the transfer of medical information at inpatient discharge [1–3], nursing home and extended care facilities (ECF) to ED transfers [4–8] as well as hand-off between residents [9], there are few studies of the transfer of data between EDs. Prior studies on inpatient discharge packets found 29.7% did not include all of the information mandated by the Joint Commission [1]. Another study found that on inpatient discharge to home care, 31% of patients had incomplete nursing paperwork [2]. While this study did not account for verbal sign-out, this does not preclude the necessity of written discharge documentation and increases the opportunity for error.

Similarly, a Cochrane review of a hospitalist to primary care physician (PCP) discharge communication found that a summary was available in only 12–34% [3]. Direct communication occurred in only 3–20%. Moreover, diagnostic test results were missing in 33–63%, treatment and hospital course in 7–22%, and tests results pending at the time of discharge were missing in 65% of cases [3].

A recent retrospective study limited to general surgery patients being transferred for surgical evaluation illustrated a lack of completeness of written communication but did not investigate the utility of transferred information [10]. Additionally, it was limited to a small subset of transfers and the retrospective review of records may not reflect real-time availability and therefore utility of the written communications. In this study, we aimed to prospectively evaluate what information is transferred between EDs across all ED-to-ED transfers and its effect on the accepting teams’ management.

## Methods

**Fig. 1:**
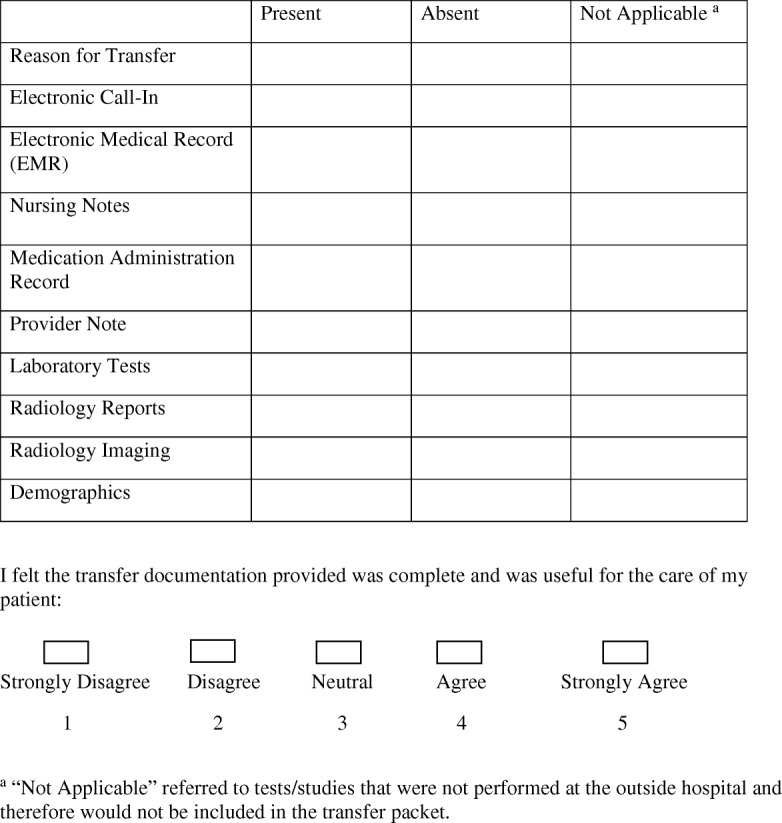
Data collection survey and utility score of transfer documentation between two emergency departments

This was a prospective, observational, and convenience sample survey study of transfer documentation conducted at an urban, academic adult tertiary medical center in Boston, MA. Transfers to our facility were initiated by the transferring facility, calling our ED, and speaking to an attending physician who subsequently accepted or declined the transfer without specific discussion regarding information to be transferred. All transfers from any ED to the study center ED were eligible for enrollment during the 2-month study period. For each ED-to-ED transfer, residents caring for the patient were asked to complete a ten-question survey about the information sent by the referring ED and available to the treating physicians at the receiving ED (Fig. 1). A “call-in” is a brief electronic summary of the patient’s illness and care at the referring facility, which is typically entered into the receiving hospital’s ED information system by the individual accepting the transfer call and linked to the patient record but is not required.

The perceived utility of the information provided was assessed using a five-point Likert scale (Fig. 1). Ratings were dichotomized into useful ≥ 4 or not useful < 4. A logistic regression analysis was performed to evaluate for which data present was most likely to result in a favorable review of the transfer documentation. This study was approved by the Institutional Review Board at our institution.

## Results

One-hundred surveys were completed. Twenty-four percent of transfers were from hospitals directly affiliated with the receiving hospital and staffed by the same physician group. The odds of receiving physicians assessing the transfer documentation as useful was increased 1.53 times, (95% CI 0.67–2.61, *p* < 0.01) if the provider note was present, and 1.39 times, (95% CI 0.58–2.44, *p* < 0.01) if the radiology report was present. These were the most commonly missing (Table 1). None of the other studied factors had a statistically significant impact on the physician-assessed usefulness of the transfer documentation. Laboratory tests and radiology imaging were not performed in three and ten cases, respectively, and were therefore marked “not applicable” (Table 1). Overall, 68% of cases had at least one component missing.

**Table 1:** Missing transfer data between two emergency departments

Category	% Missing
Electronic medical record linked to our system	87 (87/100)
Radiology report	31.1 (28/90)
Provider note	21 (21/100)
Medication administration record	20 (20/100)
Laboratory results	17.5 (17/97)
Nursing notes	15 (15/100)
Electronic call-In	14 (14/100)
Demographics	12 (12/100)
Radiology imaging	4.4 (4/90)
Reason for transfer	2 (2/100)

## Discussion

The importance of complete transfer documentation between EDs is integral to the smooth transition of care; however, as demonstrated in this study, transmission of key information is inadequate. Provider note and radiology report are viewed by physicians as the most important to aid in the transition of care; however, these are most frequently missing in the transfer documentation. The improved transition of care with appropriate documentation of medical results reduces hospital costs by avoiding redundant testing and likely helps reduce medical error [11].

Our results are similar to previous studies of hospital discharge paperwork [1, 2], as well as nursing home and ECF-to-ED transfers [4–8]. Lack of documentation has been found to increase time in ED and investigative studies in patients with altered mental status [8]. Additionally, ED length of stay (LOS) has been shown to be increased by investigative studies [12, 13] and physician hand-off [14]. For every five additional lab tests ordered, the median ED LOS increases by 10 min [12]. Moreover, lab turnaround times affect time to disposition, with every 30-min interval of lab turnaround time leading to 17 min additional LOS [12]. Compared with no testing, admitted patients with any test performed in the ED had a 49.5-min increase in LOS [13]. While our study did not look directly at LOS of transferred patients, it stands to reason that lack of complete transfer documentation may lead to repeat testing and therefore longer LOS. Furthermore, 4.4% of transferred cases did not include the radiology imaging, which may not only impact LOS but also may lead to repeat radiologic studies and unnecessary radiation exposure.

Given the similarities of findings between our study, ECF-to-ED transfers, and discharged patients to ECFs and PCPs, the question remains how best to improve the transmission of critical information during the transition of care. Gandara et al. organized wholesale changes in the umbrella corporation governing the five major hospitals in its study [1]. These included improvements to the computer-based discharge summaries to include prompts or auto-importation for required documentation, creation of discharge templates, peer review, and feedback, as well as mandated training for clinicians on proper discharge summaries [1]. While all of these methods may not be feasible in an ED setting, information technology (IT) should be at the forefront.

A standardized role of healthcare IT and electronic medical records (EMR) in hand-offs within the hospital would markedly improve safety and decrease the loss of information at patient hand-off [9]. Linking of EMRs between transferring facilities could decrease the amount of lost data. During our study period, two of the transferring facilities shared EMRs with the medical center. While 24% of cases were transferred from these institutions, EMR was available in only 13%. This may have been related to a technical delay in uploading into the EMR to be used by the accepting team or delayed linking of the patient’s information within the computer system. Both of these issues could be addressed by the IT department. Likewise, the adoption of a cloud-based radiology imaging program would ensure that all imaging is available.

**Fig. 2:**
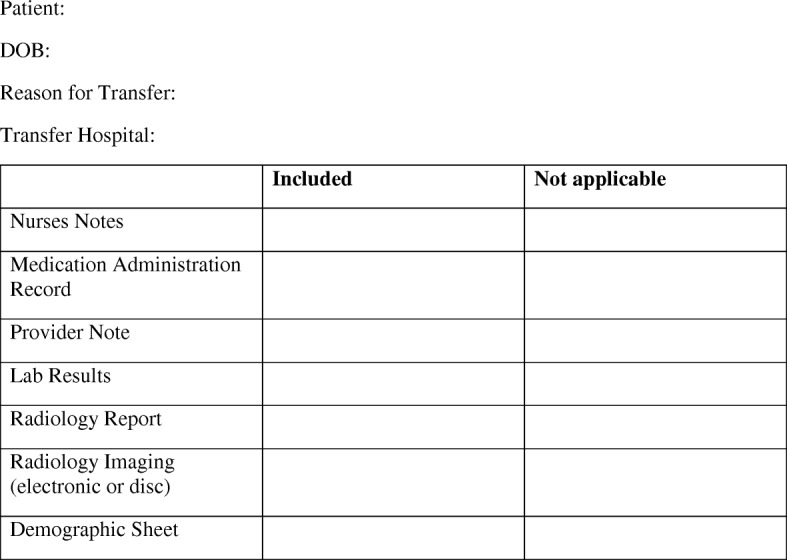
Standardized transfer packet form for ED-to-ED transfers

While it may not be feasible for an accepting facility to routinely provide feedback to the transferring facility on the quality and completeness of the transfer documentation, as implemented in Gandara et al.’s study, it is possible to create a standardized transfer checklist to be filled out by the transferring institution in order to ensure all information available is transmitted. The use of a standardized ECF-to-ED transfer sheet containing 11 essential data elements increased the amount of data provided to the ED [5]. Although it was included in only one third of transfers, it resulted in successful documentation in nearly all cases [5]. Another study developed forms for nursing home transfers, which ED staff found to be helpful in 98% of cases and more time-efficient [7]. The main critique from physicians was that the form was not always completed [7]. A standardized ED-to-ED transfer form, proposed in Fig. 2, ensuring both the provider note and radiology reports are included could improve the transfer documentation packet received by accepting facilities, thereby decreasing the time to disposition and overall LOS.

### Limitations

Our study is limited by both its small sample size and single study site. This was a convenience sample study design during which residents voluntarily filled out a survey. Multiple subjects may not have been included due to resident preference. There is no fully accurate way to determine the exact number of transfers that occurred during the 2-month study period. Inter-rater reliability was not measured between residents filling out the surveys, and it is possible that results were biased by specific individuals completing or not completing surveys. Finally, the utility score is based on a subjective decision that may not be standardized among respondents.

## Conclusions

Provider notes and radiology reports are the most useful components in transfer documentation, yet are frequently missing. Important clinical data is absent from the transfer documentation packet in 15–31% of transfers. Imaging was not included in 4.4% of transferred patients, which can lead to repeat radiologic studies and unnecessary exposure to ionizing radiation. In addition to the direct impact, this unnecessary testing may lead to increased ED LOS for transferred patients and impact overall ED LOS. There is an opportunity for significant improvement in transfer documentation, which could be aided by new IT developments allowing increased sharing of electronic records, as well as utilizing a standardized checklist to ensure transmission of valuable clinical information.

## Abbreviations

ECF: Extended care facility
ED: Emergency department
EMR: Electronic medical record
EMTALA: Emergency Medical Treatment and Labor Act
IT: Information technology
LOS: Length of stay
PCP: Primary care physician
